# Is subject-specific musculoskeletal modelling worth the extra effort or is generic modelling worth the shortcut?

**DOI:** 10.1371/journal.pone.0262936

**Published:** 2022-01-25

**Authors:** Riad Akhundov, David J. Saxby, Laura E. Diamond, Suzi Edwards, Phil Clausen, Katherine Dooley, Sarah Blyton, Suzanne J. Snodgrass

**Affiliations:** 1 Griffith Centre for Biomedical and Rehabilitation Engineering (GCORE), Menzies Health Institute Queensland, Griffith University, Brisbane, QLD, Australia; 2 School of Health Sciences and Social Work, Griffith University, Brisbane, QLD, Australia; 3 School of Health Sciences, The University of Newcastle, Callaghan, NSW, Australia; 4 Discipline of Exercise and Sport Science, Sydney School of Health Sciences, The University of Sydney, Sydney, NSW, Australia; 5 School of Environment and Life Sciences, The University of Newcastle, Callaghan, NSW, Australia; 6 School of Engineering, The University of Newcastle, Callaghan, NSW, Australia; 7 School of Allied Health, Exercise and Sports Sciences, Charles Sturt University, Bathurst, NSW, Australia; Maastricht University, BELGIUM

## Abstract

The majority of musculoskeletal modelling studies investigating healthy populations use generic models linearly scaled to roughly match an individual’s anthropometry. Generic models disregard the considerable variation in musculoskeletal geometry and tissue properties between individuals. This study investigated the physiological implications of personalizing musculoskeletal model geometry (body segment mass, inertia, joint center, and maximum isometric muscle force). Nine healthy athletes performed ten repetitions of 15 meter sprints at 75–95% of their maximum sprinting speed and ten repetitions of unanticipated sidestep cut trials with a 4.5–5.5 m/s approach running speed. Structural magnetic resonance imaging was collected on the lower extremities, from which subject-specific musculoskeletal models were developed. A one-dimensional statistical parametric mapping paired *t*-test was used to compare generic and subject-specific musculoskeletal models for: lower-limb kinematics, kinetics, torque matching, as well as hamstrings, adductors, and quadriceps muscle activations and fiber dynamics. Percentage change of geometric parameters between generic and subject-specific models were determined. Compared to generic models, subject-specific models showed significantly lower ankle dorsi/plantar flexion angle during sprinting and several significantly different net joint moments during sprint and cut tasks. Additionally, subject-specific models demonstrated better torque matching, more physiologically plausible fiber lengths, higher fiber velocities, lower muscle forces, and lower simulated activations in a subset of investigated muscles and motor tasks. Furthermore, subject-specific models identified between-limb differences that were not identified with generic models. Use of subject-specific modeling, even in healthy populations, may result in more physiologically plausible muscle fiber mechanics. Implementing subject-specific models may be especially beneficial when investigating populations with substantial geometric between-limb differences, or unilateral musculoskeletal pathologies, as these are not captured by a generic model.

## 1 Introduction

Invasive direct measurement of muscle fiber length, velocity, and force is not feasible in the study of healthy populations [1]. Musculoskeletal (MSK) modelling provides a non-invasive alternative for quantifying these, and other, biomechanical variables. An important dimension in validating an MSK model is its fidelity to an individual’s anatomy, motion, and loading. Commonly, a generic bony geometry derived from cadavers is linearly scaled to match an individual’s anthropometry based on the position of anatomical landmarks from three-dimensional motion capture [2]. This approach results in a generic MSK model with low geometric specificity [3].

In contrast, subject-specific models which mimic the individual’s musculoskeletal anatomy, derived from medical imaging (e.g., magnetic resonance imaging (MRI), computed tomography), possess high geometric specificity [4]. Depending on the level of detail in implementation, subject-specific models may include personalized body segment masses, inertia, joint definitions (e.g., centers, mechanisms), and maximum isometric muscle force generating capacity [3]. Likewise, subject-specificity may be extended to parameters outside of geometric specificity, e.g. internal muscle parameters such as tendon slack length, optimal fiber length, and pennation angle [5]. Moreover, subject-specificity may extend to the neural control approaches used to drive the MSK model [6]. Due to the cost of imaging and additional time and expertise required for analysis, the majority of MSK modelling studies investigating healthy populations use generic models, which employ mathematical optimization to predict muscle activation patterns and disregard the considerable variation in musculoskeletal geometry and tissue properties among individuals [7–9].

The use of subject-specific MSK models with high geometric specificity for subjects with musculoskeletal pathologies is recommended in the literature [3, 10]. Whether a generic scaling approach is adequate for investigating muscle tendon unit (MTU) mechanics of healthy adult locomotion is controversial. While some studies report no substantial differences in MTU mechanics between generic and subject-specific MSK models [11, 12], other studies found significantly different moment-arm and muscle-tendon lengths [13], as well as muscle and joint contact forces [14]. None of these studies accounted for subject-specific neural control of the muscles, which dominates the loading of the articulations of the lower-limb joints [15], and hence may contribute to the conflicting findings. Significantly different modelling results alone cannot elucidate whether one MSK model is a better representation of the real world subject and its motion than another. Instead, models should be compared on how closely they match in vivo experimental data, if such data are available for the specific motor task, and/or on whether model parameters are within physiologically plausible ranges.

This study compared generic and subject-specific MSK models created from the same subject group, assessed effects of geometric specificity (personalized body segment mass, inertia, joint center, and maximum isometric muscle force) on kinematics, kinetics, and MTU dynamics, and investigated the physiological implications of these effects. The primary hypotheses were that generic and subject-specific MSK models would produce significantly different kinematics, kinetics, and MTU dynamics, and that subject-specific models would result in more physiologically plausible muscle fiber mechanics.

## 2 Materials and methods

### 2.1 Participants

Nine healthy male athletes (age = 22.2±3.1 years, body mass = 86.8±20.2 kg, height = 186.0±6.0 cm) from varying competitive levels of basketball, soccer, rugby league, and rugby union were recruited for this study. Prior to data collection, approval was obtained from the institution’s Human Research Ethics Committee (H-2017-0110), and all athletes provided written informed consent.

After a five minute warm up on a stationary bicycle, participants performed a testing protocol, which consisted of a static trial (quiet upright stance) and a suite of maximum voluntary contractions using an isokinetic dynamometer (Model 770 HUMAC/NORM, Computer Sports Medicine Inc., Stoughton, MA, USA), followed by three repetitions of 15 meter sprints each performed at 100% effort, five minutes of treadmill running at 60% of maximum sprinting speed, ten repetitions of 15 meter sprints performed at 75–95% of maximum sprinting speed, five repetitions of maximum effort countermovement jumps with arm-swing, and ten repetitions of unanticipated sidestep cut trials with an approach running speed of 4.5–5.5 m/s. The suite of maximum voluntary contractions included ankle dorsiflexion, ankle plantarflexion (prone and supine), hip extension, hip abduction, hip adduction, hip flexion, hip external rotation, hip internal rotation, knee extension, and knee flexion. For each test participants completed a warm-up trial of one three-second isometric contraction against the HUMAC input arm prior to the testing protocol of three three-second isometric contractions, with one minute rest between all contractions. Participants completed all repetitions for a single testing position before moving into the next testing position, with the order standardized for all participants.

This study focuses on the submaximal sprinting and unanticipated sidestep cut tasks exclusively. Participants were wearing their own shoes but basketball shorts provided by the researchers. The sides of the shorts were taped to prevent marker occlusion. The submaximal sprinting speed and approach speed for the sidestep cut were confirmed using infrared timing gates (SmartSpeed 4-Gate System, Fusion Sport, Boulder, CO, USA). Whole foot contact on one of two ground-embedded force platforms was also required for a sprinting and/or cut task to be considered successful and suitable for analysis. Additionally, a successful cut task required a cut angle between 35° to 55°, which was marked on the floor and was visually confirmed by one researcher during each trial. Participants were given a minimum of 30 seconds rest between trials.

Two ground-embedded force platforms (2000 Hz, Type 9287CA, Kistler, Winterthur, Switzerland) connected to a control unit (Type 5695B, Kistler, Winterthur, Switzerland) were used to acquire three-dimensional GRF. Skin surface electrodes connected to a Trigno wireless EMG system (Delsys, Boston, MA, USA), sampled muscle electromyograms at 2000 Hz. Electrodes were fixed atop muscle bellies of the following 16 lower-limb muscles [16] of the participant’s dominant leg: adductor magnus, biceps femoris long head, lateral gastrocnemius, medial gastrocnemius, gluteus maximus, gluteus medius, gracilis, peroneals, rectus femoris, sartorius, semimembranosus, soleus, tensor fascia latae, tibialis anterior, vastus lateralis, and vastus medialis. At the start of testing, electrode placements were identified by a qualified clinician, reconfirmed by functional muscle test, and checked for adequate EMG signal quality [17] to ensure that EMG cross-talk was minimized. Prior to placement, the skin surface sites were shaved of hair, lightly abraded to remove dead skin, and cleansed with an alcohol swab. A 16-camera Oqus 7+ motion capture system (Qualisys AB, Gothenburg, Sweden), which sampled a whole-body retroreflective marker set [18] at 250 Hz, was used to acquire three-dimensional whole-body motion. Qualisys Track Manager (Version 2.15) was used to time synchronize and collect kinematic, kinetic, and EMG data from the motion capture system, force platforms, and EMG system, respectively. Additionally, participant’s lower extremities were imaged in the days following the data acquisition using a GE Discovery MR750w 3 T MRI unit (GE Medical Systems, Chicago, IL, USA) through a single scan of three separate stacks (sequence name: spin echo; sequence variant: segmented k-space; repetition time: 3 ms; echo time: 37.368 ms). Image sequences were optimised for bone and muscle visibility, and consisted of ~400 images with 512x512 pixel in-plane resolution, a slice thickness of 3 mm, and an inter-slice gap of 3 mm.

### 2.2 Data processing

Three-dimensional marker trajectories were labelled in the Qualisys Track Manager and exported as c3d files (C3D.org, 2020) along with GRF and EMG data. Marker trajectories and GRF were low-pass filtered with a zero-lag 4^th^ order 18 Hz Butterworth filter [19] in MATLAB (version 2019a, Mathworks, Natick, MA, USA). A zero-offset correction was performed to remove any direct current. Subsequently, EMG were band-pass filtered using a zero-lag 4^th^ order Butterworth filter (30–300 Hz), full-wave rectified, and low-pass filtered with a zero-lag 4^th^ order Butterworth filter (6 Hz) to create a linear envelope [5]. The band-pass filtered EMG signals, together with the corresponding linear envelopes, were input into the EMG classification tool [17] and EMG signal quality was assessed. All 16 resulting EMG linear envelopes were amplitude-normalized to their respective maxima obtained during the maximum voluntary contraction trials of the participant [5].

Three-dimensional representations of each participant’s bones, muscles, and outer skin boundaries were fully segmented from the MRI by one trained operator using Mimics (version 21.0, Materialise NV, Leuven, Belgium) and then visually confirmed by an expert operator. Segmented bones included the femur, tibia, and fibula form both legs, and the pelvis. In cases where the MRI did not include a full pelvis (due to the size of the athletes and fixed field of view), statistical shape modelling was used to reconstruct the pelvis from a partial segmentation [20]. Segmented muscles included: adductor group, biceps femoris long head, biceps femoris short head, lateral gastrocnemius, medial gastrocnemius, gracilis, rectus femoris, sartorius, semimembranosus, semitendinosus, soleus, vastus intermedius, vastus lateralis, and vastus medialis. Segmented outer skin boundaries included the upper and lower legs and the hip. In total, 40 objects were segmented for each participant’s MRI.

### 2.3 Musculoskeletal modelling

Two MSK models were created for each participant: a generic full-body model [21] and a subject-specific full-body model. The generic model used in this study implemented a combination of cadaver-based estimates of MTU parameters, such as optimal muscle fiber length and pennation angle, and MRI muscle volume data from 24 young healthy adults [21, 22]. The generic model was first linearly scaled in OpenSim version 3.3 [23] to match each participant’s estimated body segment dimensions, mass, and inertia. Hip joint centers were calculated using the Harrington regression equation [24]. Knee and ankle joint centers were calculated as the average of the medial and lateral markers placed atop the femoral condyles and malleoli, respectively. Calculated hip, knee, and ankle joint centers were used during the scaling procedure to improve scaling accuracy [19]. Generic fiber and tendon lengths were optimized to maintain physiological operating ranges after model scaling [25]. Maximum isometric muscle forces of the generic model were updated using the Handsfield equation, which estimates total lower-limb muscle volume and, when combined with fractional assignments to specific muscles, the volumes of individual muscles from measures of a subject’s mass and height [22].

For the subject-specific MSK model, bone segmentations were used to create a partial lower body model using the STAPLE pipeline [26]. This partial model was then combined with the upper-body portion and the feet of the generic model, updating lower body segment masses, mass centers, and hip, knee, and ankle joint centers to create the subject-specific model. To keep the whole body mass in the subject-specific model consistent, torso mass was adjusted proportional to the total difference of the lower body mass between generic and subject-specific values. Additionally, updated maximum isometric muscle forces of the segmented muscles were calculated with the following formula:

$$Maximum\;isometric\;muscle\;force=\sigma \frac{V_{m}}{l_{o}}$$

Where, *σ* is the specific muscle stress value of 60 N/cm^2^ [21], *V*_*m*_ the segmented muscle volume, and *l*_*o*_ the optimal fiber length of the muscle. Body segment inertia were calculated using outer skin boundaries and bone segmentations in nmsBuilder [27] and subsequently updated in the subject-specific model. Distance between estimated joint centers, found in the generic model, and the anatomical joint centers, used in the subject-specific model, were calculated. Personalization of the muscle attachment points and paths was not performed, as there is no consensus in the literature on how this should be implemented and adding this to our method would extend the scope of this paper beyond our intention. Thus, both generic and subject-specific models had the same muscle attachment points and paths, which were scaled from the reference model [21].

Both generic and subject-specific models for each participant were used to perform MSK modelling in OpenSim version 3.3 [23]. The lower-limb part of the models had the following degrees-of-freedom (DOF): hip and knee flexion/extension (FE), hip adduction/abduction (AA), and ankle dorsi/plantar flexion (DPF). The models included 34 MTU actuating one side of the lower-limbs [16]. Model generalized coordinates (i.e., motions), generalized loads (i.e., net joint forces and moments), and MTU kinematics (i.e., fiber lengths and moment arms) were computed in OpenSim for each trial using inverse kinematics, inverse dynamics, and muscle analysis tools, respectively. Normalized EMG linear envelopes (henceforth referred to as experimental muscle excitations), generalized loads, and MTU kinematics, were subsequently used in the Calibrated EMG-informed Neuromusculoskeletal Modelling Toolbox (CEINMS) [6]. Here, 30% of both the cut task and sprinting trials with the best EMG quality were selected for calibration. Calibration adjusted generic values of tendon slack length, optimal fiber length, pennation angle, activation dynamics parameters, and maximum isometric force of each muscle of the participant’s generic and subject-specific models in a subject-specific manner, while constrained to physiological ranges, to minimize error in joint moments and experimental EMG [6]. Maximum isometric muscle forces of previously segmented muscles used in the subject-specific model were not adjusted by CEINMS.

After each participant’s model was calibrated, CEINMS was used in EMG-assisted mode [6] to predict muscle lengths, velocities, and forces during the cut tasks and the sprint. The EMG-assisted mode optimized existing excitations determined from experimental EMG signals to better match experimental joint moments by the use of two weighting factors that define how much muscle excitations and joint moments can deviate from their respective experimental data. For muscles with no experimental EMG, or with EMG quality classified as unusable by the EMG classification tool [17], CEINMS implemented static optimization to predict muscle excitations [28]. CEINMS was configured to use an elastic tendon with the root finding method for musculotendon dynamics. Both models used subject-specific MTU parameters derived through CEINMS calibration and a subject-specific neural solution calculated with CEINMS in EMG-assisted mode, as this study solely focuses on the effects of geometric specificity.

**Fig 1:**
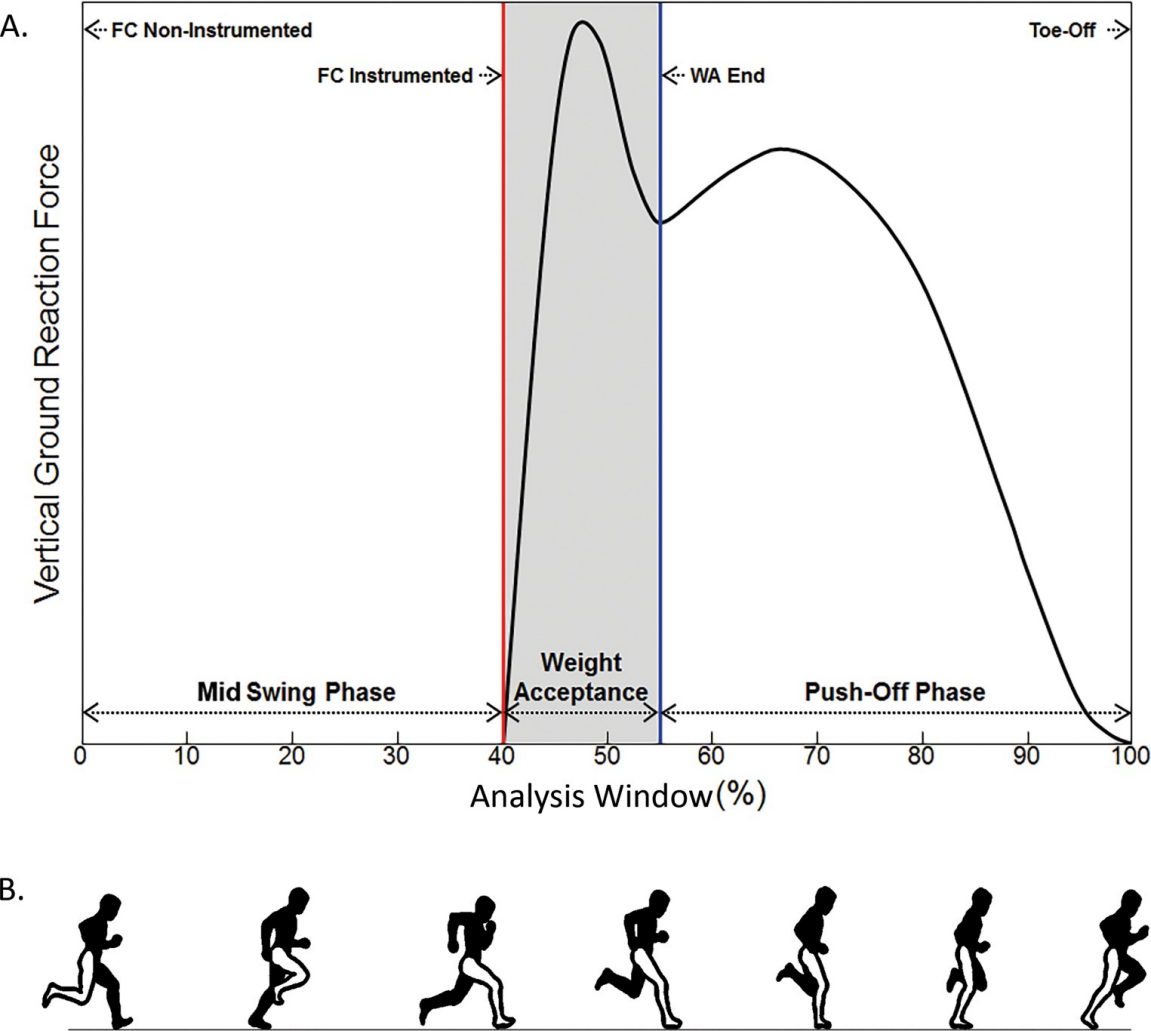
Visualization of the ground reaction force at corresponding phases of the stride cycle. (A.) Temporal events: foot-ground contact of the non-instrumented leg (FC Non-Instrumented), foot-ground contact of the instrumented leg (FC Instrumented), end of weight acceptance phase (WA End), and toe-off (Toe-Off). Analysis Window (%) starts with FC Non-Instrumented and ends with Toe-Off of the instrumented leg. (B.) Instrumented leg depicted in white. Phases of the stride cycle correspond to the Analysis Window (%) in Fig 1A.

Time continuous biomechanical data were normalized to 101 frames in total consisting of three distinct temporal phases: swing and stance phases, with the latter subdivided into weight acceptance and push-off (Fig 1). Analysis started in ipsilateral swing with the non-instrumented (i.e., contralateral) foot in contact with the ground, which was visually confirmed. Foot-ground contact of the instrumented leg, detected by the vertical GRF exceeding 20 N, indicated the end of ipsilateral swing and the start of weight acceptance. End of weight acceptance and start of push-off was defined by the first local minimum of the vertical GRF (Fig 1). Toe-off (the end of push-off and the analysis) was defined by vertical GRF falling under a threshold of 20 N. The analysis window tracked the majority of a stride cycle with 0–40% of the analysis window representing swing, initial foot-ground contact, and start of weight acceptance; the end of weight acceptance occurred at 55% of the analysis window, and 55–100% of the analysis window representing push-off and final foot-ground contact. Furthermore, mid stance was defined as the first half of push-off (55.0%-77.5% of the analysis window). Each muscle’s fiber length and velocity were normalized by the muscle-specific optimal fiber length and maximum shortening velocity, respectively. Muscle forces were also normalized to the respective subject-specific body weight, but this did not alter the study’s results, thus original muscle forces were reported.

### 2.4 Statistical analyses

To verify the CEINMS calibration process, coefficient of determination (R^2^) and root-mean-square error (RMSE) were calculated for hip FE, hip AA, knee FE, and ankle DPF moments predicted by CEINMS and those from inverse dynamics. Of the 34 MTU dynamics estimated by CEINMS, four hamstrings (biceps femoris long head (BFLH), biceps femoris short head (BFSH), semimembranosus (SM), and semitendinosus (ST)), four adductors (adductor brevis (AB), adductor longus (AL), adductor magnus (AM), and gracilis (GRA)), and four quadriceps muscles (rectus femoris (RF), vastus intermedius (VI), vastus lateralis (VL), and vastus medialis (VM)) were statistically analyzed. These muscle were selected because their muscle borders were clearly visible on the MRI images and could be segmented with high accuracy. All four compartments of AM in the participant’s model were analyzed, combined, and reported as AM.

Statistical parametric mapping (SPM) was used to compare muscle lengths, velocities, forces, and activations, as well as inverse kinematics and inverse dynamics of the generic and the subject-specific models [29]. A one-dimensional SPM paired t-test (α = 0.05) was used [29]. All SPM analyses were implemented using the open-source spm1d code (v.M.0.4.7, www.spm1d.org) in MATLAB (version 2019a, Mathworks, Natick, MA, USA). For geometric parameters (body segment mass, inertia, joint center, and maximum isometric muscle force), percentage change between the generic and subject-specific models was calculated.


$$Percentage\;change=\frac{SM-GM}{GM}*100\%$$

Where, SM is the subject-specific model and GM is the respective generic model. Additionally, within-subject percentage change in peak muscle forces and activations between generic and subject-specific models were calculated and reported in the S1 and S2 Tables.

## 3 Results

### 3.1 Changes in model parameters

Average body segment masses differed between generic and subject-specific models (Table 1). In subject-specific models, pelvis and lower leg segment masses were 18.1–21.0% lower, whereas the upper leg and torso masses were 5.3–10.5% higher. Segment inertia for pelvis and lower leg was 14.7–40.4% lower (Table 1). Segment inertia of the upper leg increased by 0.3–4.9% about x and z axes and decreased by 7.1–9.6% about the y-axis.

**Table 1:** Body segment mass and inertia changes between generic and subject-specific models.

	Pelvis	Upper R	Upper L	Lower R	Lower L	Torso
**GM (kg)**	13.8 ±3.3	10.9 ±2.6	10.9 ±2.6	4.4 ±1.0	4.4 ±1.0	31.5 ±7.4
**SM (kg)**	11.3 ±2.6	12.1 ±3.7	11.8 ±3.7	3.5 ±0.8	3.4 ±0.8	33.2 ±7.2
**Change (%)**	-18.1 ±3.2	10.5 ±6.4	7.9 ±7.1	-20.4 ±6.4	-21.0 ±6.4	5.3 ±4.4
**xx (%)**	-25.7	4.5	4.9	-38.5	-39.6	
**yy (%)**	-14.7	-7.1	-9.6	-33.3	-34.8	
**zz (%)**	-26.3	0.7	0.3	-39.3	-40.4	

Upper, upper leg; Lower, lower leg; R, right side of model; L, left side of model; GM, generic model; SM subject-specific model; Change, percentage change SM/GM; xx, yy, and zz, percentage change SM/GM for moment of inertia of body segment about corresponding axis of body frame. Pelvis, Upper, Lower, and Torso in kilogram ±standard deviation.

Anatomical joint centers, implemented in the subject-specific model, differed from the ones estimated by the generic model. Euclidean distance between anatomical and generic joint centers differed at the hip (right 2.3±0.4 cm, left 2.3 ±0.3 cm), knee (right 2.5 ±1.0 cm, left 2.6 ±1.0 cm), and ankle (right 1.0 ±0.7 cm, left 1.1 ±0.4 cm). Subject-specific maximum isometric forces of all analyzed muscles were 12.7–43.7% higher compared to corresponding generic values (Table 2). Inter-limb (i.e., left compared to right) differences in maximum isometric muscle force, ranging from -3.9% to 2.2%, were only identified in the subject-specific model.

**Table 2:** Maximum isometric muscle forces in generic and subject-specific models.

	Hamstrings				Adductors				Quadriceps			
	BFLH	BFSH	SM	ST	AB	AL	AM	GRA	RF	VI	VL	VM
**R&L GM (N)**	1396 ±209	602 ±115	2351 ±357	630 ±100	663 ±106	970 ±143	657 ±102	300 ±48	2334 ±346	1837 ±351	5562 ±1068	2966 ±571
**R SM (N)**	1623 ±220	865 ±160	2663 ±653	859 ±110	924 ±176	1353 ±249	916 ±172	399 ±71	3024 ±411	2418 ±402	6688 ±1046	3448 ±778
**L SM (N)**	1601 ±197	845 ±132	2679 ±658	854 ±141	914 ±153	1338 ±214	906 ±150	408 ±65	2906 ±323	2361 ±371	6515 ±977	3342 ±643
**R SM/GM (%)**	16.3	43.7	13.3	36.3	39.4	39.4	39.4	33.0	29.6	31.6	20.2	16.3
**L SM/GM (%)**	14.7	40.4	13.9	35.4	38.0	37.9	38.0	36.0	24.5	28.5	17.1	12.7
**L/R SM (%)**	-1.3	-2.3	0.6	-0.6	-1.0	-1.1	-1.0	2.2	-3.9	-2.3	-2.6	-3.1

BFLH, biceps femoris long head; BFSH, biceps femoris short head; SM, semimembranosus; ST, semitendinosus; AB, adductor brevis; AL, adductor longus; AM, adductor magnus; GRA, gracilis; RF, rectus femoris; VI, vastus intermedius; VL, vastus lateralis; VM, vastus medialis; R, right side of model; L, left side of model; GM, generic model; SM subject-specific model; SM/GM, percentage change SM/GM; L/R, percentage change L/R of SM. Maximum isometric muscle forces in Newton ±standard deviation.

### 3.2 Modelling results

**Fig 2:**
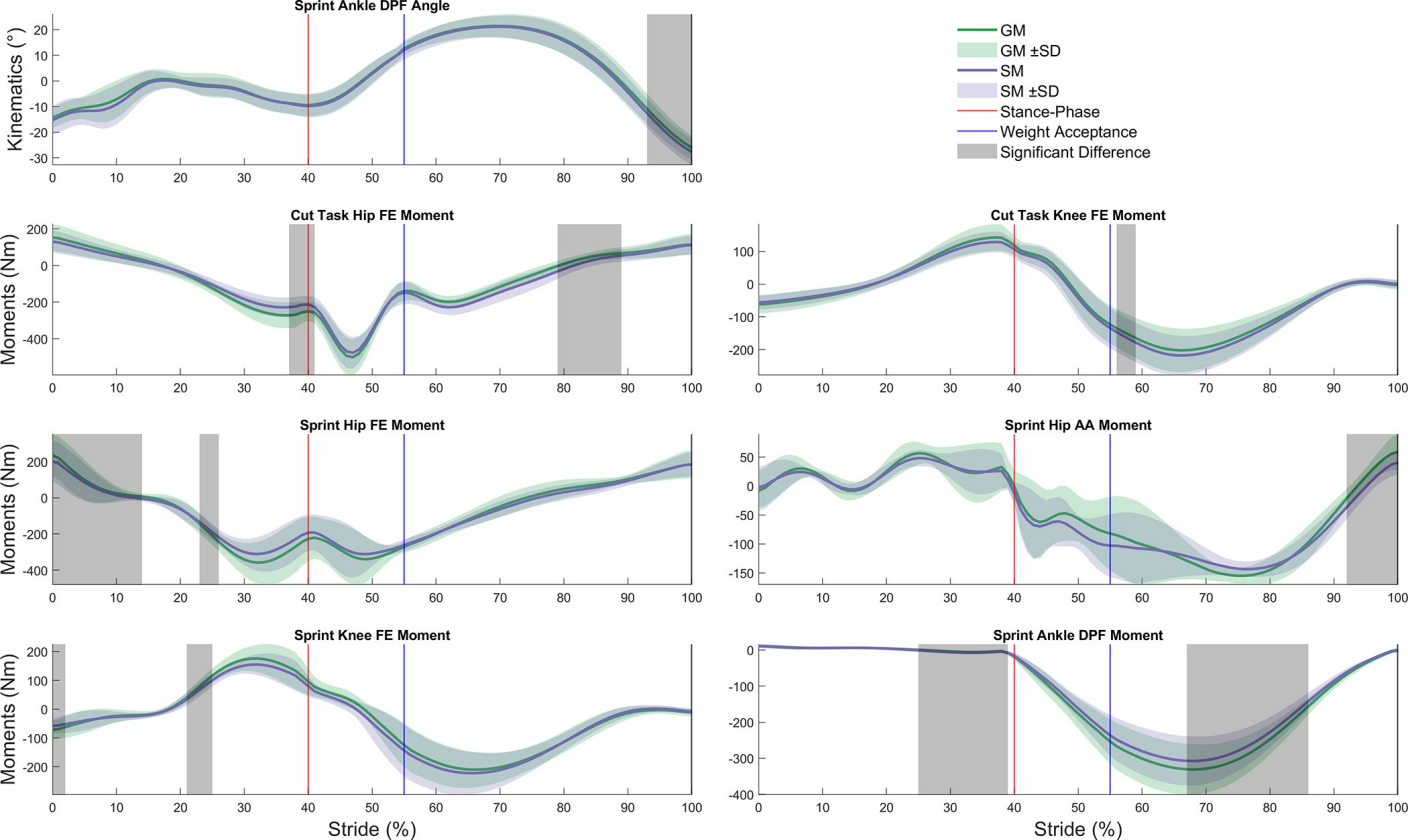
Significant joint angle and moment differences between generic and subject-specific models. FE, flexion/extension; AA, adduction/abduction; DPF, dorsi/plantar flexion; Generic model (GM), subject-specific model (SM), and respective variation between participants as standard deviation. Foot ground contact of the stance limb depicted as the red line. End of weight acceptance phase depicted as the blue line. Areas of significant difference, as determined by statistical parametric mapping (grey).

**Fig 3:**
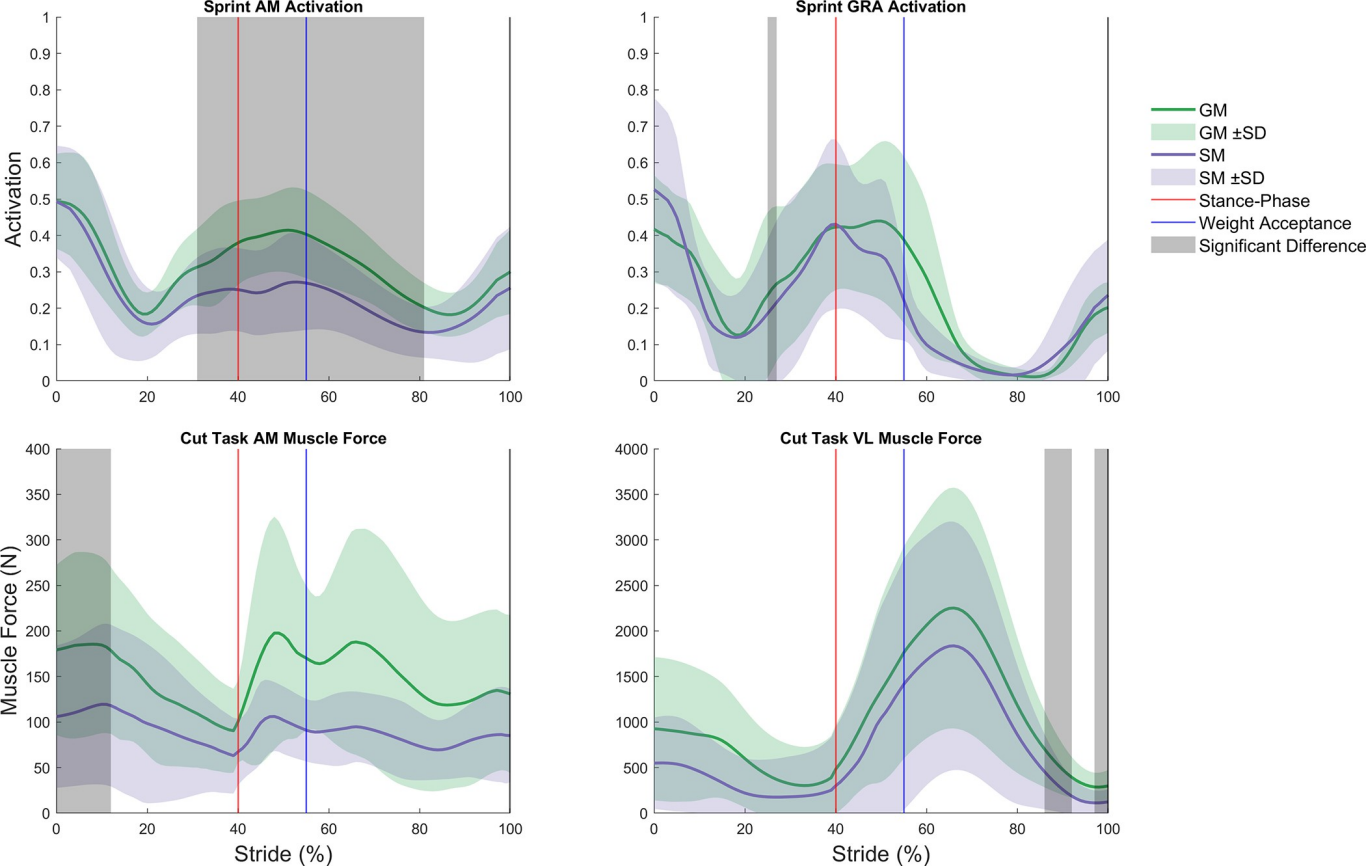
Significant activation and muscle force differences between generic and subject-specific models. AM, adductor magnus; VL, vastus lateralis; Generic model (GM), subject-specific model (SM), and respective variation between participants as standard deviation. Foot ground contact of the stance limb depicted as the red line. End of weight acceptance phase depicted as the blue line. Areas of significant difference, as determined by statistical parametric mapping (grey).

The CEINMS calibration achieved joint moments with R^2^ of 0.78–0.96 and RMSE of 8.5–49.6 Nm/kg for all DOF compared to external joint moments determined through inverse dynamics (Table 3) for both models. Compared to generic models, all R^2^ were higher (0.6–8.8%) and all RMSE were lower (1.0–52.1%) for subject-specific models. A statistically significant between-model difference in kinematics was found exclusively for ankle DPF (9.8% lower in SM, p = 0.008) during the sprint (Fig 2). No other model DOF during sprint or cut tasks showed significant between-model kinematic differences. Compared to generic models, subject-specific models displayed significantly higher hip FE moments (15.2% difference, p = 0.039) around foot-ground contact, lower hip FE moments (24.1% difference, p = 0.002) during weight acceptance, and lower knee FE moments (8.5% difference, p = 0.037) during the cut task (Fig 2). During sprinting, subject-specific models displayed significantly lower hip FE moments (42.2% difference, p = 0.001) during mid swing, higher hip FE moments (8.4% difference, p = 0.033) during late swing, lower hip AA moments (54.4% difference, p<0.001) during push-off, higher knee FE moments (17.0% difference, p = 0.041) during mid swing, lower knee FE moments (14.1% difference, p = 0.026) during late swing, lower ankle DPF moments (38.0% difference, p<0.001) during late swing, and higher ankle DPF moments (7.9% difference, p<0.001) during mid stance (Fig 2). No significant between-model differences in CEINMS-adjusted muscle activations were found during the cut task, but AM (34.2% difference, p<0.001) and GRA (20.5% difference, p = 0.049) exhibited significantly lower activations in the subject-specific model during the sprint (Fig 3).

**Table 3:** Matching of joint moments predicted by CEINMS to those from inverse dynamics for generic and subject-specific models.

	Cut				Sprint			
	Hip FE	Hip AA	Knee FE	Ankle DPF	Hip FE	Hip AA	Knee FE	Ankle DPF
**GM R** ^**2**^	0.92 ±0.05	0.86 ±0.07	0.96 ±0.03	0.93 ±0.02	0.88 ±0.07	0.78 ±0.04	0.89 ±0.07	0.93 ±0.04
**SM R** ^**2**^	0.98 ±0.03	0.92 ±0.04	0.96 ±0.03	0.95 ±0.02	0.92 ±0.08	0.85 ±0.07	0.92 ±0.04	0.94 ±0.04
**Change (%)**	5.9 ±3.1	7.8 ±4.4	0.6 ±0.2	2.1 ±1.7	4.8 ±3.2	8.8 ±5.4	3.6 ±1.1	1.8 ±0.6
**GM RMSE (Nm/kg)**	34.2 ±10.8	12.1 ±4.2	17.3 ±5.5	24.0 ±7.7	49.6 ±13.1	26.7 ±6.8	33.0 ±6.1	19.4 ±5.9
**SM RMSE (Nm/kg)**	16.4 ±13.7	8.5 ±3.2	15.9 ±7.2	18.6 ±9.6	29.6 ±14.2	18.6 ±4.8	24.5 ±1.7	19.2 ±9.5
**Change (%)**	-52.1 ±19.0	-29.6 ±9.1	-8.3 ±3.7	-22.3 ±11.6	-40.3 ±16.0	-30.2 ±12.4	-25.9 ±7.3	-1.0 ±0.2

FE, flexion/extension; AA, adduction/abduction; DPF, dorsi/plantar flexion; GM, generic model; SM subject-specific model; Change, percentage change SM/GM; R^2^, coefficient of determination ±standard deviation; RMSE, root-mean-square error in Nm/kg ±standard deviation.

**Fig 4:**
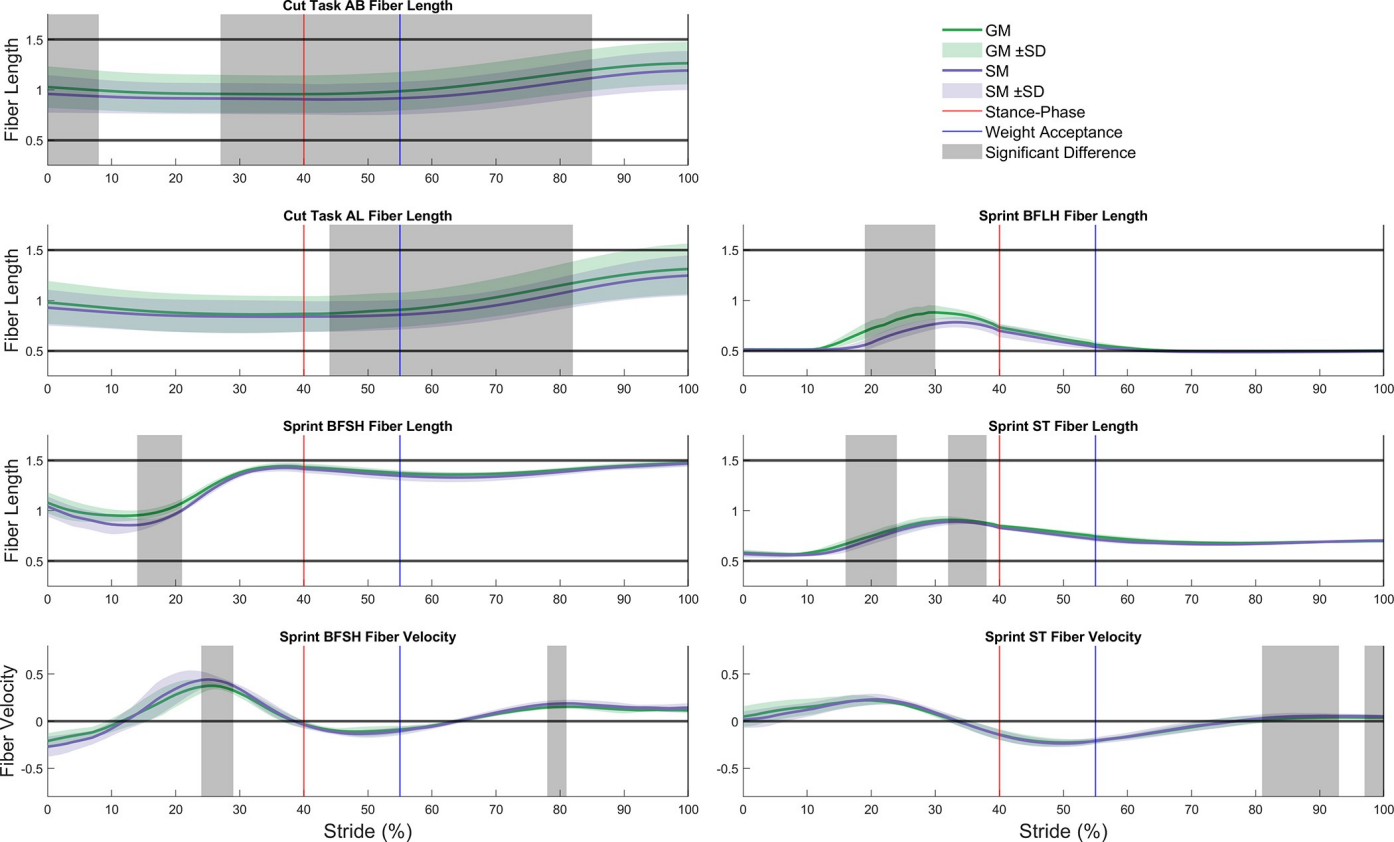
Significant normalized fiber length and velocity differences between generic and subject-specific models. AM, adductor brevis; AL, adductor longus; BFLH, biceps femoris long head; BFSH, biceps femoris short head; ST, semitendinosus; Generic model (GM), subject-specific model (SM), and respective variation between participants as standard deviation. Foot ground contact of the stance limb depicted as the red line. End of weight acceptance phase depicted as the blue line. Areas of significant difference, as determined by statistical parametric mapping (grey). Each muscle’s fiber length was normalized by the respective optimal fiber length and each muscle’s fiber velocity was normalized by the respective maximum shortening velocity.

Compared to generic models, subject-specific models produced significantly lower force in AM (38.1% difference, p = 0.010) and VL (43.3% difference, p = 0.032 and 60.0% difference, p = 0.044) during the cut task, but no differences during the sprint. Compared to generic models, subject-specific models exhibited significantly shorter normalized fiber lengths in AB (6.3% difference, p = 0.049 and 6.7% difference, p = 0.008) and AL (6.3% difference, p = 0.005) during the cut task and in BFLH (16.3% difference, p = 0.012), BFSH (8.7% difference, p = 0.043), and ST (4.6% difference, p = 0.008 and 1.8% difference, p = 0.024) (Fig 4) during the sprint. In generic models, normalized fiber lengths in four muscles (AB = 1.56, AL = 1.60, GRA = 1.58, and VL = 1.60) exceeded physiological ranges of 0.50 to 1.50 [30] during the cut task and in two muscles (AL = 1.57 and GRA = 1.56) during the sprint. In contrast, subject-specific models had no muscle fiber lengths outside physiological ranges for either task. No significant between-model differences in normalized fiber velocity were found during the cut task, whereas BFSH (16.6% difference, p = 0.032 and 22.1% difference, p = 0.031) and ST (52.6% difference, p<0.001 and 33.7% difference, p = 0.042) velocities were significantly higher in the subject-specific compared to generic models during the sprint.

## 4 Discussion

This study investigated differences in model estimates of muscle and joint mechanics resulting from inclusion of high geometric specificity and the implications of these differences in healthy adults. Anatomically derived parameters including body segment mass, inertia, joint center, and maximum isometric muscle force, which were included in subject-specific models, differed from the generic scaled estimations. Indeed, compared to generic models the inclusion of high geometric specificity in a subject-specific model led to significant differences in a subset of modelled kinematics and joint moments and resulted in better torque matching, physiologically plausible fiber lengths, higher fiber velocities, lower muscle forces, and lower simulated activations. Six out of ten observed regions of significant difference in joint moments were found in the swing phase (three in hip FE, two in knee FE, and one in ankle DPF), which may be the phase of risk for hamstring injury during sprinting [31]. If feasible, subject-specific MSK modelling is worth the extra resources and effort, and may be necessary even when investigating non-pathological populations if confidence in the modelling outputs is desired. Implementing subject-specific models may be especially beneficial when studying populations with unilateral musculoskeletal pathologies, as these are not captured by a generic model.

Compared to anatomically-derived model parameters, generic models overestimated pelvis and lower leg segment masses by 18.1–21.0% and underestimated upper leg and torso segment masses by 5.3–10.5%. These differences in physical properties of the segments were also seen in segment inertia. Generic bones were uniform and generally bigger than imaging-derived bones, meaning they accounted for a higher percentage of segment volume (e.g., femur within thigh segment). Additionally, the generic model underestimated maximum isometric muscle forces, a product of the respective muscle volume adjusted by CEINMS calibration, by 12.7–43.7%. Even though the generic model chosen for this study implemented muscle volume estimates from young healthy adults [21], these estimates might not be representative of an athletic population, which could explain the observed discrepancies. Overall, the discrepancy between approximated and real-world bone-to-muscle ratios of the body segments could have contributed to the observed significant differences in kinematics, joint moments, and MTU mechanics between the generic and the subject-specific models.

Between-limb differences in the maximum isometric muscle force were only identified in the subject-specific model. The right leg was dominant in all but two participants and showed, on average, bigger muscle volumes measured from MRI, resulting in higher maximum isometric muscle forces compared to the left leg. This between-limb difference is not captured in the generic model and could be particularly relevant in populations with unilateral musculoskeletal pathologies. Anatomical joint centers, implemented in the subject-specific model, were located higher and more anterior for the right hip (distance of 2.3±0.4 cm), lower and more anterior for the right knee (distance of 2.5 ±1.0 cm), and lower for the right ankle (distance of 1.0 ±0.7 cm) with minimal between-limb differences. Previous studies have shown changes in hip joint center location can not only affect simulated muscle activation of the hip abductors and hip contact force by up to three times body weight [32], but also lower the muscle forces that contribute to hip joint torques [3, 33]. Thus, the inclusion of subject-specific joint centers, as well as between-limb differences in the maximum isometric muscle force, could have further contributed to the significant differences in MTU mechanics between the generic and the subject-specific models observed in this study.

The subject-specific model implemented body segment mass, inertia, joint centers, and maximum isometric muscle force that were anatomically derived and thus are likely to be more physiologically plausible than those estimated by the generic model. Muscle fiber lengths of four muscles during the cut task (AB = 1.56, AL = 1.60, GRA = 1.58, and VL = 1.60) and two muscles during the sprint (AL = 1.57 and GRA = 1.56) were found to be outside the physiologically plausible range [30] in the generic model exclusively. To the best of our knowledge, no *in vivo* experimental data of adductor, quadriceps, or hamstring MTU dynamics for the sprint and/or the cut task are available to date. Consequently, confirmatory statements about which model is more physiologically plausible are difficult, since for example joint moments of both models are within ranges that can be generated statically on controlled machinery.

Limitations of the results presented in this paper should be considered. Bone, muscle, and outer skin boundary segmentations and the anatomical parameters derived from those (segment mass, segment inertia, joint centers, and maximum isometric muscle forces) have small uncertainties and inter-operator variabilities which could have affected the results. To reduce these uncertainties, the segmentations were completed by a single operator, confirmed by another trained operator, and measures were applied consistently across subject-specific models using a codified workflow [26]. Additionally, to keep the whole body mass in the subject-specific model consistent with the participant’s mass, torso mass was adjusted with the resulting difference of the lower body mass, but torso inertia remained identical to the generic model since the upper body was not captured in the MRI. A full-body MRI would enable upper body segmentations and further increase geometric specificity of the subject-specific model. An additional limitation was that a sensitivity evaluation of model parameters responsible for the observed differences between generic and subject-specific models was not performed, as such an analysis was outside the scope of this study. This study included nine participants; a larger cohort may detect further changes in some of the parameters investigated. Personalization of the muscle attachment points and paths was not performed, as there is no consensus in the literature on how this should be implemented. The role of personalized muscle attachment points and paths in modulating modelling outputs is likely substantial and hence should be investigated further. The newly discovered role of titin in muscle force production [34] was not considered in this study, as Hill-type muscle models do not currently include titin in their formulation. Furthermore, only sprinting and cutting, movements integral to many sports, were investigated in this study. Subject-specific geometry might influence different sets of movement tasks uniquely. Lastly, these results should be interpreted with caution, since *in vivo* experimental data for the investigated muscles and motor tasks is not yet available.

## 5 Conclusions

In conclusion, subject-specific models with anatomically derived geometric parameters showed significantly lower ankle dorsi/plantar flexion angle during sprinting and several significantly different net joint moments during both sprinting and cutting compared to generic models. These between-model differences resulted in better torque matching, more physiologically plausible fiber lengths, higher fiber velocities, lower muscle forces, and lower simulated activations for the subject-specific model in a subset of muscles. Therefore, it is recommended that studies of healthy subjects implement subject-specific modeling over generic modelling to generate more physiologically plausible MTU mechanics. Additionally, implementing subject-specific models may be especially beneficial when investigating populations with substantial geometric between-limb differences, or unilateral musculoskeletal pathologies, as these are not captured by a generic model.

## Supporting information

S1 Table Comparison of average CEINMS adjusted muscle activation between the generic and the subject-specific models. BFLH, biceps femoris long head; BFSH, biceps femoris short head; SM, semimembranosus; ST, semitendinosus; AB, adductor brevis; AL, adductor longus; AM, adductor magnus; GRA, gracilis; RF, rectus femoris; VI, vastus intermedius; VL, vastus lateralis; VM, vastus medialis; GM, generic model; SM subject-specific model; Change, percentage change SM/GM.
(DOCX)

S2 Table Percentage change of maximum muscle force between the generic and the subject-specific models of individual participants. BFLH, biceps femoris long head; BFSH, biceps femoris short head; SM, semimembranosus; ST, semitendinosus; AB, adductor brevis; AL, adductor longus; AM, adductor magnus; GRA, gracilis; RF, rectus femoris; VI, vastus intermedius; VL, vastus lateralis; VM, vastus medialis; Values in percent (subject-specific model/generic model).
(DOCX)

## Abbreviations

AA: adduction/abduction
AB: adductor brevis
AL: adductor longus
AM: adductor magnus
BFLH: biceps femoris long head
BFSH: biceps femoris short head
CEINMS: calibrated electromyography-informed neuromusculoskeletal modelling toolbox
DOF: degrees of freedom
DPF: dorsi/plantar flexion
EMG: electromyography
FE: flexion/extension
GRA: gracilis
GRF: ground reaction forces
MRI: magnetic resonance imaging
MSK: musculoskeletal
MTU: muscle tendon unit
R^2^: coefficient of determination
RF: rectus femoris
RMSE: root-mean-square error
SM: semimembranosus
SPM: statistical parametric mapping
ST: semitendinosus
VI: vastus intermedius
VL: vastus lateralis
VM: vastus medialis
