# Supplementary material for: Is subject-specific musculoskeletal modelling worth the extra effort or is generic modelling worth the shortcut?
Source: PLoS One. 2022 Jan 25;17(1):e0262936. doi: 10.1371/journal.pone.0262936 (PMC8789151; doi:10.1371/journal.pone.0262936)
Supplement: S1 Table — BFLH, biceps femoris long head; BFSH, biceps femoris short head; SM, semimembranosus; ST, semitendinosus; AB, adductor brevis; AL, adductor longus; AM, adductor magnus; GRA, gracilis; RF, rectus femoris; VI, vastus intermedius; VL, vastus lateralis; VM, vastus medialis; GM, generic model; SM subject-specific model; Change, percentage change SM/GM. (DOCX) [file pone.0262936.s001.docx]

**S1 Table.** **Comparison of average CEINMS adjusted muscle activation between the generic and the subject-specific models.**

|  | **Hamstrings** | | | | **Adductors** | | | | **Quadriceps** | | | |
| --- | --- | --- | --- | --- | --- | --- | --- | --- | --- | --- | --- | --- |
|  | **BFLH** | **BFSH** | **SM** | **ST** | **AB** | **AL** | **AM** | **GRA** | **RF** | **VI** | **VL** | **VM** |
| **GM Cut Task** | 0.69 | 0.55 | 0.77 | 0.64 | 0.20 | 0.17 | 0.44 | 0.54 | 0.33 | 0.79 | 0.63 | 0.81 |
| **SM Cut Task** | 0.49 | 0.50 | 0.73 | 0.61 | 0.23 | 0.17 | 0.27 | 0.57 | 0.39 | 0.71 | 0.51 | 0.79 |
| **Change (%)** | -27.9 | -9.5 | -6.0 | -5.0 | 11.4 | -2.5 | -39.6 | 4.4 | 17.9 | -10.3 | -19.4 | -2.0 |
| **GM Sprint** | 0.79 | 0.56 | 0.79 | 0.62 | 0.35 | 0.27 | 0.41 | 0.44 | 0.26 | 0.60 | 0.61 | 0.54 |
| **SM Sprint** | 0.72 | 0.63 | 0.82 | 0.64 | 0.23 | 0.15 | 0.27 | 0.43 | 0.33 | 0.64 | 0.68 | 0.58 |
| **Change (%)** | -8.2 | 11.4 | 3.8 | 3.6 | -32.7 | -44.4 | -34.4 | -2.0 | 27.5 | 6.2 | 11.3 | 7.7 |

BFLH, biceps femoris long head; BFSH, biceps femoris short head; SM, semimembranosus; ST, semitendinosus; AB, adductor brevis; AL, adductor longus; AM, adductor magnus; GRA, gracilis; RF, rectus femoris; VI, vastus intermedius; VL, vastus lateralis; VM, vastus medialis; GM, generic model; SM subject-specific model; Change, percentage change SM/GM.
