# Supplementary material for: Is subject-specific musculoskeletal modelling worth the extra effort or is generic modelling worth the shortcut?
Source: PLoS One. 2022 Jan 25;17(1):e0262936. doi: 10.1371/journal.pone.0262936 (PMC8789151; doi:10.1371/journal.pone.0262936)
Supplement: S2 Table — BFLH, biceps femoris long head; BFSH, biceps femoris short head; SM, semimembranosus; ST, semitendinosus; AB, adductor brevis; AL, adductor longus; AM, adductor magnus; GRA, gracilis; RF, rectus femoris; VI, vastus intermedius; VL, vastus lateralis; VM, vastus medialis; Values in percent (subject-specific model/generic model). (DOCX) [file pone.0262936.s002.docx]

**S2 Table.** **Percentage change of maximum muscle force between the generic and the subject-specific models of individual participants.**

|  | **Cut Task** | | | | | | | | | | | |
| --- | --- | --- | --- | --- | --- | --- | --- | --- | --- | --- | --- | --- |
|  | **Hamstrings** | | | | **Adductors** | | | | **Quadriceps** | | | |
|  | **BFLH** | **BFSH** | **SM** | **ST** | **AB** | **AL** | **AM** | **GRA** | **RF** | **VI** | **VL** | **VM** |
| **1** | -59.0 | 33.4 | -35.5 | -35.4 | 0.6 | -26.8 | -27.4 | -75.7 | -26.2 | 34.4 | -22.6 | 20.1 |
| **2** | -93.1 | -4.2 | 44.8 | 46.1 | 8.7 | 12.4 | -26.8 | -6.2 | -18.3 | 55.8 | 6.0 | 4.1 |
| **3** | -95.7 | 75.8 | 13.8 | 6.4 | -59.8 | -16.6 | -70.4 | 58.3 | 34.3 | -1.7 | -55.4 | -22.2 |
| **4** | 284.4 | -68.0 | -71.6 | 511.5 | -21.1 | -27.0 | -41.2 | 88.0 | -93.9 | 9.7 | -47.9 | -0.4 |
| **5** | -44.7 | 15.8 | 18.7 | 170.2 | -31.1 | -19.4 | -61.8 | -88.1 | -66.3 | 44.4 | 27.4 | 123.2 |
| **6** | -37.6 | 26.8 | -52.7 | -43.8 | -30.3 | -30.0 | 8.2 | 36.5 | -31.8 | 18.5 | 20.5 | 12.1 |
| **7** | -95.3 | 142.2 | -50.3 | 17.7 | 38.1 | 79.6 | -28.4 | -49.5 | -69.8 | -64.7 | -65.9 | 83.0 |
| **8** | -36.1 | 32.0 | -56.8 | 139.6 | -14.3 | -15.1 | -21.7 | -20.4 | -56.0 | 1.7 | -46.1 | -0.5 |
| **9** | 4.9 | -43.1 | -13.0 | -37.5 | -0.3 | -17.8 | -20.5 | -39.1 | 29.5 | -17.0 | -33.2 | -11.5 |
|  | **Sprint** | | | | | | | | | | | |
| **1** | -2.2 | -56.8 | -36.1 | -0.2 | 6.6 | 17.0 | 19.3 | 28.0 | -31.1 | 33.4 | 8.8 | 30.0 |
| **2** | -88.1 | 56.1 | 35.1 | -49.7 | 26.9 | 24.3 | 34.7 | 9.1 | 18.4 | 50.6 | -19.2 | 19.1 |
| **3** | 46.4 | -39.8 | -43.1 | -48.3 | -37.6 | -48.1 | -34.6 | -79.9 | -34.3 | -46.7 | -46.1 | -68.3 |
| **4** | -27.7 | 211.3 | -31.3 | -68.2 | 5.9 | 26.7 | 34.8 | 54.2 | -13.8 | 90.0 | 22.3 | 32.6 |
| **5** | -26.6 | 198.3 | -37.6 | -7.7 | -12.7 | 16.6 | 0.1 | 87.1 | -13.3 | 75.3 | 18.8 | -10.1 |
| **6** | -79.9 | 27.4 | -27.4 | 26.1 | 120.3 | 59.2 | -11.8 | -39.1 | -8.1 | -35.2 | -31.1 | 98.6 |
| **7** | -76.3 | 20.9 | -35.0 | 0.0 | -33.0 | -44.6 | -31.4 | 18.1 | 23.6 | -27.8 | -35.1 | 137.9 |
| **8** | 12.7 | -46.3 | -46.2 | 84.3 | -46.1 | -52.1 | -29.3 | -82.3 | -44.0 | -40.1 | -40.8 | -65.9 |
| **9** | 47.7 | -8.3 | -47.9 | -2.8 | 11.6 | -14.4 | 83.2 | -6.9 | 140.8 | 16.9 | 7.8 | 37.4 |

BFLH, biceps femoris long head; BFSH, biceps femoris short head; SM, semimembranosus; ST, semitendinosus; AB, adductor brevis; AL, adductor longus; AM, adductor magnus; GRA, gracilis; RF, rectus femoris; VI, vastus intermedius; VL, vastus lateralis; VM, vastus medialis; Values in percent (subject-specific model/generic model).
